# Interventions to Improve the Cast Removal Experience for Children and Their Families: A Scoping Review

**DOI:** 10.3390/children8020130

**Published:** 2021-02-10

**Authors:** Pramila Maharjan, Dustin Murdock, Nicholas Tielemans, Nancy Goodall, Beverley Temple, Nicole Askin, Kristy Wittmeier

**Affiliations:** 1 Physiotherapy Department, Health Sciences Centre Winnipeg–Shared Health, Winnipeg, MB R3A 1R9, Canada; pmaharjan@hsc.mb.ca (P.M.); ngoodall@hsc.mb.ca (N.G.); 2 Orthopedic Technology Services, Health Sciences Centre Winnipeg–Shared Health, Winnipeg, MB R3A 1R9, Canada; 3 Department of Physical Therapy, College of Rehabilitation Sciences, Rady Faculty of Health Sciences, University of Manitoba, Winnipeg, MB R33 0T6, Canada; murdoc22@myumanitoba.ca (D.M.); umtielem@myumanitoba.ca (N.T.); 4 College of Nursing, Rady Faculty of Health Sciences, University of Manitoba, Winnipeg, MB R3T 2M6, Canada; bev.temple@umanitoba.ca; 5 WRHA Virtual Library, University of Manitoba, Winnipeg, MB R3E 3P5, Canada; nicole.askin@umanitoba.ca; 6 Department of Pediatrics and Child Health, Rady Faculty of Health Sciences, University of Manitoba, Winnipeg, MB R3A 1S1, Canada; 7 Children’s Hospital Research Institute of Manitoba, Winnipeg, MB R3E 3P4, Canada

**Keywords:** pediatric, cast removal, anxiety, cast room, patient experience, fracture, scoping review

## Abstract

Background: Cast removal can be a distressing experience for a child. This scoping review aims to provide a comprehensive review of interventions designed to reduce anxiety and improve the child’s and family’s experience of pediatric cast removal. Methods: A scoping review was conducted (Medline, Embase, PsycINFO, CINAHL, Scopus, grey literature sources). Inclusion criteria: studies published January 1975–October 2019 with a primary focus on pediatric patients undergoing cast removal/cast room procedures. Screening, full text review, data extraction, and quality appraisal were conducted in duplicate. Results: 974 unique articles and 1 video were screened. Nine articles (eight unique studies) with a total of 763 participants were included. Interventions included the following, alone or in combination: noise reduction, electronic device use, preparatory information, music therapy, play therapy, and child life specialist-directed intervention. Heart rate was used as a primary (88%) or secondary (12%) outcome measure across studies. Each study reported some positive effect of the intervention, however effects varied by age, outcome measure, and measurement timing. Studies scored low on outcome measure validity and blinding as assessed by the Joanna Briggs Institute Critical Appraisal Checklist for Randomized Controlled Trials. Conclusion: Various methods have been tested to improve the pediatric cast removal experience. Results are promising, however the variation in observed effectiveness suggests a need for the use of consistent and valid outcome measures. In addition, future research and quality improvement projects should evaluate interventions that are tailored to a child’s age and child/family preference.

## 1. Introduction

Anxiety related to cast removal has been discussed in the medical literature for nearly 100 years. In 1922, Dr. Clarence E. Rees noted: *“The removal of heavy plaster casts is practically always a tedious task for both surgeon and patient; the one wearing out his hands with the exertion of cutting and the other his nervous system with the expectation of being cut”* [1] (p. 147). This may be especially relevant in the pediatric setting. In general, children undergoing medical procedures may feel afraid or anxious due to feelings of powerlessness, a perceived lack of control, a lack of knowledge about the procedure, or previous memories of a painful and distressing health care experience [2,3,4]. Ensuring a safe and positive experience for children during medical procedures is important for the immediate experience and has long-term implications. Positive or negative medical interactions can shape perceptions toward health care and health care-related behaviours for life [5].

Accordingly, recommendations have been developed to help parents and health care providers prevent or reduce a child’s experience of pain or distress during specific types of painful or invasive health care procedures and in primary care settings [6,7]. One approach to framing recommendations is the “3P’s” approach, which categorizes strategies to prevent or reduce pain or distress during medical procedures as physical, psychological or pharmacologic [8,9]. Examples of physical approaches include infant or child positioning, and parent presence or participation during the procedure [8]. Psychological techniques can include relaxation, preparatory strategies, or distraction [8,9]. Pharmacologic approaches may include a topical anesthetic or mild sedation [8]. Tailoring the approaches to the age of the child, as well as the procedure are important, and guidance documents have provided clear recommendations for procedures such as needle pokes, laceration repair, and surgery [8,9]. Cast removal has not been specifically addressed within these guidance documents to date.

Cast removal has unique procedural elements that may trigger or worsen anxiety such as the fear of being cut and the noise produced by the cast removal system [10]. This, along with longstanding awareness of anxiety related to cast removal [1] highlights the need for evidence-based recommendations that are tailored to the procedure. The purpose of this scoping review was to provide a comprehensive review of the literature to identify and document strategies that have been specifically developed and evaluated to reduce anxiety and improve the child’s and family’s experience during pediatric cast removal. 

## 2. Materials and Methods

A scoping review was conducted to map the current literature, summarize the available evidence, and identify research gaps on the topic of improving children’s experience and reducing anxiety related to cast removal [11,12,13]. A protocol was developed a priori. The initial search strategy was developed by the medical librarian team member, revised with input from team members, and peer reviewed by a second medical librarian (Appendix A). The search strategy was adapted for use with Medline, Embase, PsycINFO, CINAHL, Scopus databases, PEDro, Trip, ISRCTN, clinicaltrials.gov, ICTRP, Google Scholar (results of first 10 pages reviewed), and Google (results of first 5 pages reviewed). The strategy was implemented in October 2019. Research team members included practicing physiotherapists who are trained and experienced in cast removal (PM, NG), a medical librarian (NA), two clinician researchers (BT, KW), and two physiotherapy students (DM, NT).

### 2.1. Inclusion/Exclusion Criteria

Initial inclusion criteria for published and grey literature included (i) publication date between January 1975 and October 2019, (ii) primary focus on pediatric patients (≤18 years) undergoing cast removal, (iii) published in English language, (iv) conducted with humans, and (v) including a measure of family experience (either parent, child, or both; author defined and including but not limited to satisfaction, anxiety, distress, level of preparedness etc.). During preliminary screening and in keeping with the flexibility afforded by scoping review [11,13], a team decision was made to retain studies that examined strategies to reduce a child’s anxiety during other tasks performed in the cast room (e.g., cast application, pin or suture removal). Results that dealt primarily with the Ponseti method or backslab casts were excluded as these represent a specific casting technique, require a different removal technique, or involve patient populations that limit relevance to the general cast removal experience. 

Search results were uploaded into Rayyan, a web-based tool, to facilitate sorting and screening of the literature [14]. Screening was performed by two research team members (DM, NT) who independently reviewed all available abstracts of peer-reviewed literature and grey literature to determine eligibility for inclusion. Full texts were obtained if both reviewers agreed on inclusion, if discrepancy existed, or if abstracts were not available. The two reviewers then independently reviewed full texts. Conflicts were discussed between the reviewers and if agreement was not reached a third team member (KW) was available to help achieve consensus regarding inclusion.

### 2.2. Data Extraction

The two reviewers extracted and charted data from the included literature using a modified version of the TIDeR (Template for Intervention Description and Replication) checklist; a reporting tool developed to assist with comprehensive reporting of an intervention [15]. Fields were modified by merging with guidance from scoping review methods papers [11,12,13]. Data extraction was verified by two additional authors (PM, KW). Interventions were broadly classified into two of the “3P’s” categories for pain management; physical, and psychological [8,9]; with pharmacologic strategies being outside the scope of this review. The unique strategies employed within each category were further identified where able (e.g., distraction, preparatory information etc.) [8].

### 2.3. Critical Appraisal

A critical appraisal of the studies included in the scoping review was conducted using the Joanna Briggs Institute Critical Appraisal Checklist for Randomized Controlled Trials [16]. This peer-reviewed tool has been developed to analyze the quality of research included in reviews, including the degree to which research teams have addressed the risk of bias within the individual studies [16]. Each included study was critically appraised by two team members (DM, KW) using the Joanna Briggs Institute Checklist and corresponding manual [16]. 

## 3. Results

**Figure 1:**
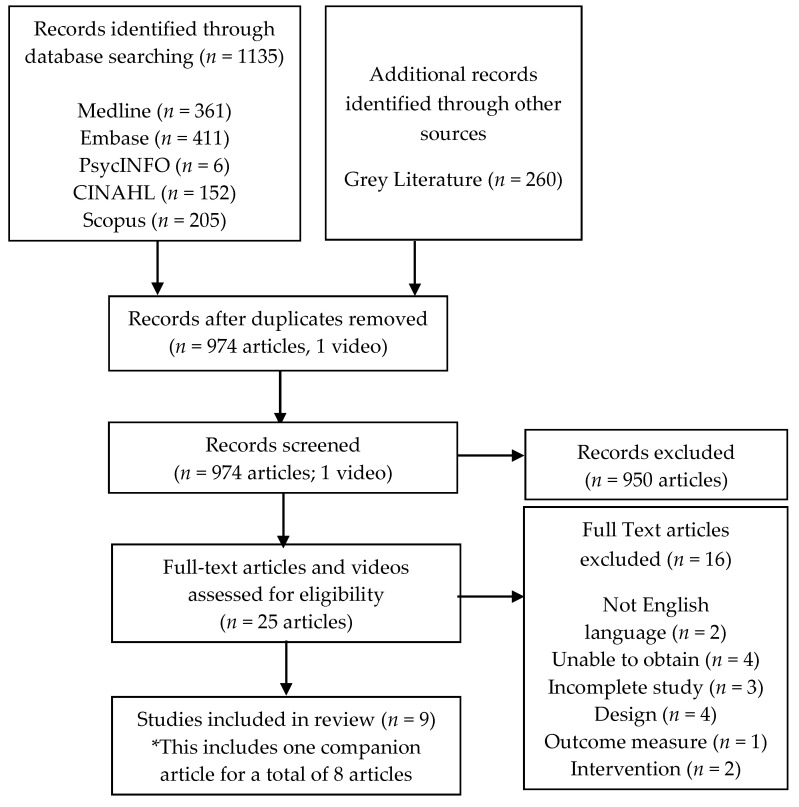
Preferred Reporting Items for Systematic Reviews and Meta-Analyses (PRISMA) scoping review diagram.

After removing duplicates, 974 articles and 1 video were retrieved and screened for review. Nine articles, representing eight unique studies were ultimately included (Figure 1). As per inclusion criteria, study participants were children and youth 0–18 years of age, undergoing cast removal or cast room procedures (e.g., cast application, pin removal, fracture reduction etc.; Table 1). Study sample sizes ranged from 20–208 participants (median = 85) for a total of 763 participants across the 8 unique studies. 

### 3.1. Interventions

The 8 included studies evaluated the impact of interventions that included noise reduction [17,20,23], the inclusion of other specialists (play therapist, child life therapist) [24,25], electronic tablets for distraction [21], preparatory sensation vs. procedural-based information [18,19], and music therapy [22] (Table 2). Two interventions outlined the use of theory in developing the intervention [18,19,25]. When categorized using the 3P’s approach, two studies investigated the impact of a physical intervention; specifically noise reduction [17,20]. Five evaluated psychologically-based interventions [18,19,21,22,24,25]; employing the strategies of distraction [21,24,25], preparatory information [18,19], music therapy [22], and play [24,25], alone or in combination. One study used both physical and psychological approaches, combining the strategies of noise reduction and distraction (Table 2) [23]. 

### 3.2. Outcomes

All studies had a primary focus on the patient’s experience during the procedure, using either a physiologic or behavioral response to measure anxiety. Heart rate (HR) was used across all studies either as primary (88%) or secondary (12%) outcome measure of anxiety/distress. One study used mean arterial pressure in addition to HR to measure anxiety [17]. Four (50%) of the included studies used additional non-physiological outcomes to measure anxiety, including observed behaviour or behavioural scales [18,19,23,24,25]. Three studies measured parent satisfaction or experience [23,24,25]. One study measured the satisfaction of the cast room clinician [25] (Table 3). 

Each study reported a positive effect of the intervention under study, however effects varied by age group, outcome measure, and timing of measurement (Table 3). Noise reduction was found to be effective in reducing anxiety during cast removal (measured by HR) when evaluated alone [17,20], or in combination with use of an electronic device [23], although results varied by age [17] (Table 3). The provision of sensory-based information was also effective to reduce anxiety during cast removal; although the child’s level of fear about the procedure was an important mediating variable [18,19]. Therapeutic play had favourable effects on anxiety during cast removal, although the significance again varied by age group, as well as by outcome measure [25]. The impact of the certified child life specialist (CCLS) intervention was reported to have favourable outcomes when using a non-validated study-specific behavioural scale, but not when assessed with HR [24]. The use of an iPad with video was associated with a lower HR prior to but not during cast room procedures, while the use of an iPad with video games increased HR at most time points [21]. Adverse events associated with interventions were documented in two studies [17,20]. One study reported that four younger children (ages 3–6 years) refused to wear earphones/cried until they were removed [17], and the other study reported no adverse events [20].

### 3.3. Critical Appraisal

**Figure 2:**
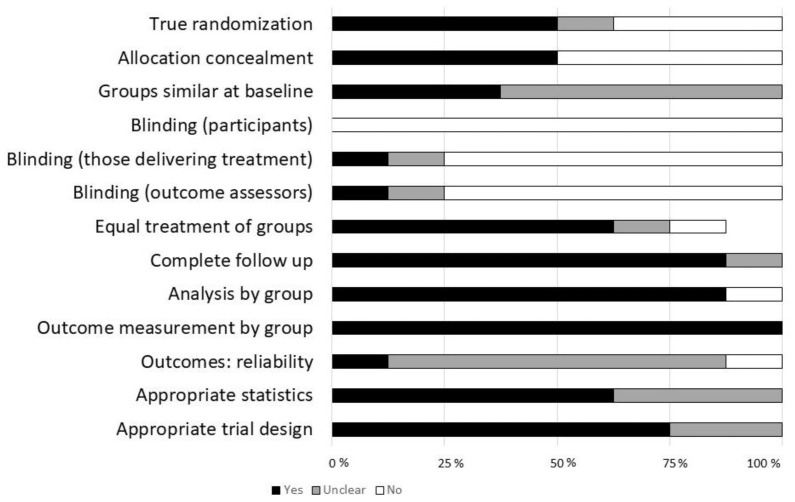
Critical assessment of included studies. Critical appraisal completed using Joanna Briggs Institute critical appraisal tool; checklist for randomized controlled trials [16]. Color key: black = yes; grey = unsure; white = no.

Thirteen categories are assessed within the Joanna Briggs Institute Critical Appraisal Checklist for Randomized Controlled Trials (Figure 2) [16]. Studies consistently measured outcomes by initial grouping. Most studies however scored “unclear” for outcome measure validity, and due to the nature of the interventions blinding was often impractical or difficult to achieve. Of the 13 categories assessed with the checklist, one study met the requirements in 10/13 categories, one met 8/13, four met 7/14, one met 4/13 and one met 3/13.

## 4. Discussion

This scoping review examined literature that evaluated interventions to reduce anxiety in children undergoing cast removal or other procedures conducted in the cast room. Eight unique studies were included in this review, evaluating physical strategies (noise reduction), psychological strategies (preparatory information, distraction, music therapy, play), and a combined physical and psychological approach (noise reduction and distraction). While some benefit was found with each intervention, results varied by age group, level of fear prior to the procedure, outcome measure, and timing of measurement. 

A limitation across the studies included in this review is the use of HR, sometimes exclusively, to quantify the distress or anxiety experienced by the participant during the cast room procedure. While HR has been shown to correlate with anxiety, it is not always an accurate measure [31], and does not exclusively measure anxiety which may confound results. For example, HR increased in the intervention group using video games [21]. It would be reasonable to expect that this could be a physiologic response to involvement with the video game rather than anxiety. Also, while the use of behavioural scales or other measures may help overcome limitations of HR as a measure of anxiety, there was limited use of validated behavioural measures. Incongruent results between HR and other measures of distress or anxiety make it difficult to assess the true impact of certain interventions [25]. Large age ranges within participant groups [17,21], and the inclusion of a range of cast room procedures [21,24] are other potential confounders within the included studies that may have limited the power to detect the effect of an intervention at different time points, or within age or procedure sub-groups. Non-intervention factors may have also confounded results in certain studies. For example, in the study evaluating CCLS intervention, it was reported that a television was often playing in the background of the clinic rooms [24]. The authors identify that this, and the insufficient recording of CCLS modalities used during the intervention, may have impacted their findings [24]. 

Despite limitations, the evidence summarized within this review can be used to inform future research or quality improvement work. For example, certain interventions appeared to be more effective for select age ranges. A pragmatic intervention approach would be to have evidence-informed options available, along with information for families regarding which strategies might be most useful for certain age groups. For example, offering the use of soft music for infants and children who may not tolerate headphones [17,22], noise-cancelling headphones for older children/those who tolerate them [17,20], and the option to use personal electronic devices for those who choose headphones [23]. Similarly, level of fear could also be regularly assessed prior to cast room procedures to help identify children who may benefit most from strategies to reduce anxiety [18,19]. Research and quality improvement teams should work together with orthopedic clinic staff to leverage staff member’s experience in calming children during procedures, to co-develop processes for routine assessment of baseline fear or anxiety, and to share evidence with families regarding approaches and techniques that can be used to reduce anxiety during cast room procedures. Working with clinic staff can help to ensure consistent, feasible, and sustainable approaches to anxiety reduction. Importantly, research and quality improvement teams should also work with parents and children to design interventions, and to determine when and how to share information about strategies that can be used to improve the cast removal experience. Involving and evaluating the involvement of clinicians, patients, and family members in research and quality improvement initiatives is recommended to accomplish improvements in health care [32,33,34], yet was not discussed in any of the studies included. Finally, there is a growing body of research and guidance around the prevention of pain or distress in children undergoing health care procedures [6,7,8,9]. Several techniques (e.g., parental presence, comfort positioning, non-nutritive sucking [8]) recommended for other procedures were not evaluated in the studies included in this review. Future work could consider including and evaluating the effectiveness of these or other strategies as appropriate, to reduce anxiety during cast room procedures. 

### Study Limitations

Limitations of this review include the potential for missed relevant literature due to database selection, search criteria, and limitation to English language. We attempted to mitigate the risk of missed literature by having the search strategy developed by a medical librarian and peer reviewed by a second medical librarian. Including grey literature further enhanced the comprehensiveness of this review. Consistent with the methods of a scoping review, no attempt was made to combine the data for meta-analysis. Although heart rate was used in all studies, the differences in measurement time points, variety of interventions tested, and inclusion of additional cast room procedures are factors that would limit pooling of data. Further research and future meta-analysis could strengthen recommendations for practice change. 

## 5. Conclusions

This study provided a review of interventions to reduce anxiety associated with pediatric cast removal or cast room procedures. In general, both physical (noise reduction) and psychological (interventions using distraction, preparatory information, music therapy, play) interventions demonstrated some beneficial effect to reduce anxiety although this varied with age, outcome measure, baseline fear, and time point of measurement. Most of the interventions reviewed here are relatively easy to implement, however additional evaluation of these interventions alongside other 3P techniques will better inform clinicians on the best use of resources to improve children’s experience in the cast room. Ideally, future research and quality improvement projects will be co-designed by families, clinicians, and researchers to develop locally tailored, effective, and sustainable solutions. 

## Figures and Tables

**Table 1:** Study characteristics.

Reference (First Author [ref])	Year, Country	Study Site	Age of Participants (Range)	Sample Size (Male/Female)	Procedure(s) During Which Intervention Was Tested
Carmichael [17]	2005; US	Not specified	0–18	100 (62/48)	Cast removal
Johnson [18,19]	1975 and 1976; US	Children’s hospital, orthopedic fracture clinic	6–11	84 (52/32)	Cast removal
Katz [20]	2001; Israel	Not specified	5–6	20 (not specified)	Cast removal, forearm fracture
Ko [21]	2016; US	Orthopedic clinic	1–18	146 (84/62)	Cast room procedures (cast removal, cast placement, cast overwrap, splint placement, fracture reduction, joint injection, dressing change, suture removal, aspiration, external fixator structural change, pin removal)
Liu [22]	2007; US	Hospital orthopedic clinic	0–10	69 (not specified)	Cast room procedures (cast removal, cast application, pin removal, suture removal)
Mahan [23]	2017; US	Outpatient orthopedic clinic	2–10	50 (32/18)	Cast removal
Schlechter [24]	2017; US	Orthopedic clinic	2–10	86 (not specified)	Cast room procedures (splint removal, initial cast placement, fracture manipulation, pin removal, cast removal)
Wong [25]	2017; China	Regional teaching hospital, orthopedic outpatient clinic	3–12 Randomization stratified by age (3–7, 8–12)	208 (135/73)	Cast removal

Abbreviations: US (United States of America).

**Table 2:** Description of interventions.

Study (First Author, [Ref])	Why (Rationale/Theory, Goal)	What (Intervention (I); Control (C))	Possible Physical/Psychological Strategies Involved ^1^	How It Was Implemented (Mode; When and How Much)	Who Provided	Tailoring
Physical						
Carmichael [17]	Rationale: Noise and vibration of saw likely contribute to anxiety during cast removal. This may be improved with ear protection. Goal: Study the impact of hearing protection on anxiety in children of different age groups.	I: Noise reducing hearing protection during cast removal. C: No hearing protection.	Noise reduction	Hearing protection was given to children prior to starting cast removal procedure.	NR	NR
Katz [20]	Rationale: A serious adverse event had occurred during cast removal at the study author’s institution. Goal: To address a gap in the literature on anxiety and anxiety reduction during cast removal in children.	I: Noise reducing hearing protection during cast removal C: No hearing protection.	Noise reduction	Hearing protection was given during cast removal.	NR	NR
Psychological						
Johnson [18,19]	Theory: Informed by work linking cognitive processes and emotions/ behaviour (Lazarus, RS [26]; Schachter and Singer [27]), as well as author’s previous research demonstrating impact of interactions tailored to patient’s emotional needs. Builds on authors own research related to preparatory sensory-based information. Piaget’s theory of cognitive development informed the participant age inclusion criteria [28]. Goal: To affect the emotional response to cast removal through cognitive processes.	I: Recorded description of sensations during cast removal. C1: Recorded description of procedure of cast removal. C2: No recording.	Preparatory information	Children heard recordings (via headphones) prior to procedure.	Study nurse	NR
Ko [21]	Rationale: Electronic devices had been found to reduce anxiety during other health care procedures but had not been evaluated during cast room procedures. Goal: Determine if use of iPads reduce anxiety during cast removal.	I1: iPad with video I2: iPad with game. C: No iPad	Distraction	iPads were given to the patients as they went into the cast room to use during the procedure.	NR	Intervention group participants could choose from a list of available videos or games.
Liu [22]	Rationale: Anxiety and other adverse events can occur during cast removal. Hearing protection may be beneficial but not all children can wear headphones. Music therapy has demonstrated some effectiveness in other settings. Goal: Study the impact of soothing music in cast room with children.	I: Background lullaby music in the cast room clinic. C: No music.	Music therapy	Cast rooms were randomized to music or no music; music was playing when participant entered room.	Not applicable	NR
Schlechter [24]	Rationale: CCLS have been found to improve children’s health care experience in certain settings, but the role and impact of CCLS within the cast room had not been previously described/studied. Goal: Describe the impact of a CCLS in the cast room setting	I: Certified Child Life Specialist (CCLS) present. C: No CCLS present.	Preparatory information; play; distraction	CCLS present and interacting with child during procedure.	CCLS	CCLS used variety of methods (not documented consistently).
Wong [25]	Theory: Theory of stress and coping (Lazarus and Coleman [29]). Theory of cognitive development (Piaget [30]) used to determine age ranges. Rationale: Therapeutic play to improve a child’s sense of control during cast room procedures may assist with coping and reduce anxiety. Goal: To study the effect of therapeutic play on anxiety for children undergoing cast removal.	I: Preparation and distraction play plus standard care. C: Standard care (nurse provided preparatory information; reassurance)	Preparatory information; play; distraction	Preparation play was delivered before cast removal; distraction play during procedure.	Hospital play specialist	Play methods tailored to child’s preferences; parent presence and involvement supported.
Combined physical & psychological						
Mahan [23]	Rationale: Loud cast saws can contribute to anxiety in children during cast removal; and anxiety may lead to behaviours that can result in injury (e.g., pulling away). Using headphones combined with a device has not been studied. Goal: Reduce exposure to noise to reduce anxiety during cast removal and improve parent satisfaction.	I: Noise cancelling headphones with electronic device (music/video, videogames). C: Standard care	Noise reduction; distraction	Headphones were used before, during and after cast removal.	Not specified	Families were asked to bring own device with child’s preferred media; could also borrow device from facility.

Abbreviations: CCLS = Certified Child Life Specialist, I = intervention, C = control, NR = not reported. ^1^ Techniques informed by 3P Framework as outlined in Trottier et al. [8].

**Table 3:** Outcome measures and findings.

Study (First Author [Ref])	Outcome Measures	Findings
Physical		
Carmichael [17]	Heart Rate (HR) Mean arterial pressure (MAP)	Age combined data: less increase in HR in intervention vs. control (8.4% vs. 14.4%; *p* = 0.04). Non-significant (NS) differences MAP. Age stratified data: non-significant trend toward smaller increase in HR with intervention in younger children (<13 y). NS difference MAP. Author’s conclusion: Using hearing protections appeared to have a positive effect on HR but not MAP. Younger children appear to benefit more.
Katz [20]	HR	Less increase in HR intervention vs. control (11.1% vs. 26.9%; *p* =< 0.01) Author’s conclusion: Hearing protection reduced anxiety during cast removal as measured by HR.
Psychological		
Johnson [18,19]	Stick figure score (fear) Observed and scored behaviour (distress) HR	Fear prior to cast removal was associated with distress scores across all groups (*p* < 0.001). Fear prior to cast removal was associated with fear during the procedure (*p* < 0.001). Participants in the sensation-based information group had lower distress scores than the no information control group (*p* < 0.025). Significant increase in HR (from waiting room to during procedure) in control group and procedure-based intervention group; NS increase in HR in the sensation-based intervention group. Author’s conclusion: Sensation-based preparatory messages can reduce distress during cast removal; baseline fear level is an important factor in fear and distress during the procedure.
Ko [21]	HR	Higher HR in intervention group (video game) before procedure. Decrease in HR from waiting room to before procedure in intervention group (video) for all procedures, and when cast removal analysed separately (*p* < 0.05), however increase in HR in intervention (video) group during procedure (*p* = 0.047). Author’s conclusion: Using an iPad with video may assist with lowering HR before the procedure, but the sound of the cast saw during the procedure may eliminate this benefit.
Liu [22]	HR	A more favourable change in HR from the waiting room to procedure room in intervention group, across all procedures (−2.7 bpm vs. 4.7 bpm. *p* = 0.001) and in sub-group analysis (cast removal or cast application). A more favourable change in HR for the intervention group was also observed from waiting room to during procedure, across procedures (15.3 bpm vs. 22.5 bpm; *p* = 0.05); no differences observed when analysed by sub-groups. No difference in change in HR between groups at other time points (before intervention to during intervention; before intervention to after intervention). Author’s conclusion: Soft lullaby music may improve young children’s experience in the cast room, when entering the cast room and during procedures.
Schlechter [24]	HR Behavioural scale (study specific) Parent survey	NS differences in HR between groups. More favourable behavioural score in intervention group (*p* < 0.01). NS difference in parent surveys (experience, child behaviour) between groups. Author’s conclusion: Certified Child Life Specialist presence seems to positively effect cast room experience based on the study-specific behavior scale that was used.
Wong [25]	Visual Analogue Scale-Anxiety (VAS-A): Ages 3–7 years Chinese version of the State Anxiety Scale for Children (CSAS-C): Ages 8–12 years Children’s Emotional Manifestation Scale (CEMS) (emotional behavior) Satisfaction scale (parent; technician) HR	VAS-A, ages 3–7 years: Significant difference in change in anxiety (before procedure to after), favouring intervention group (*p* = 0.010). CSAS-C, ages 8–12 years: NS difference between intervention and control. CEMS: Emotional behaviour during the cast removal favoured intervention groups (*p* < 0.001). Parent and cast technician satisfaction higher with intervention group (*p* = 0.02; *p* < 0.001 respectively). Stratified by age group: NS difference between groups for 3–7 years old; significantly lower HR in intervention group before and during procedure for 8–12 year-olds (*p* = 0.037). Author’s conclusion: Therapeutic play can reduce anxiety and improve experience (including parent and technician satisfaction) of cast removal.
Combined (physical and psychological)		
Mahan [23]	Faces, Legs, Activity, Cry, Consolability scale (FLACC) HR Parent anxiety and satisfaction	NS differences in FLACC before or after procedure between groups; lower FLACC during procedure in intervention group (*p* = 0.03). Lower HR before (*p* = 0.02) and after the procedure (*p* = 0.005) in intervention group. Older age was associated with lower HR before, during and after procedure across groups, and lower FLACC scores during procedure. No differences in parent anxiety or satisfaction between groups. Author’s conclusion: Headphones and device use can improve children’s anxiety during cast removal.

Abbreviations: CEMS = Children’s Emotional Manifestation Scale, CSAS-C = Chinese version of the State Anxiety Scale for Children, FLACC = Faces, Legs, Activity, Cry, Consolability scale, HR = heart rate, MAP = mean arterial pressure, NS = non-significant, VAS-A = Visual Analogue Scale-Anxiety.

## Data Availability

No new data were created or analyzed in this study. Data sharing is not applicable to this article.

## Appendix A

**Table A1:** Search strategy for Medline Ovid, run October 2019.

exp infant/or exp child/or adolescent/
child health/or infant health/or adolescent health/
exp child health services/or adolescent health services/
exp pediatrics/or exp pediatricians/or exp nurses, pediatric/or pediatric nurse practitioners/or pediatric assistants/or adolescent medicine/or hospitals, pediatric/or exp pediatric nursing/
(p?ediatric*).tw,kf
(infant* or infancy).tw,kf
(baby* or babies).tw,kf
(neonat* or newborn* or new-born*).tw,kf
(child* or kid or kids).tw,kf
(schoolchild* or school age* or schoolage* or primary school* or elementary school* or secondary school* or high school* or highschool*).tw,kf
(preschool or pre-school or toddler or kindergar* or nursery).tw,kf
(adoles* or teen* or youth or youths or young people or young person* or young adult* or pre-teen* or preteen*).tw,kf
(boy* or girl*).tw,kf
or/1–13
exp Anxiety/or exp fear/or stress, psychological/or frustration/
exp Anxiety Disorders/or stress disorders, traumatic/or psychological trauma/or stress disorders, post-traumatic/or stress disorders, traumatic, acute/or exp phobic disorders/
exp patient satisfaction/
(anxiet* or stress disorder* or psychological* trauma* or ptsd or ptsds or post trauma* or posttrauma* or (stress* adj2 trauma*)).tw,kf
(anxious* or fear or fears or fearful* or nervous* or worrie* or worry* or angst or apprehensi* or panic* or phobic or phobia* or distress* or stress* or uncomfortable or discomfort* or frustrat* or unhapp*).tw,kf
((patient* or parent* or family or child*) adj3 (experience* or satisf* or preparedness or dissatisf*)).tw,kf
or/15–20
casts, surgical/
((fiberglass or fibreglass or cast or casts or casting or plaster* or spica) adj5 (remov* or room or clinic or saw or saws or sawing or cut or cuts or cutting)).tw,kf
((cast or casts or casting or orthopedic* or fracture) adj3 (technician* or technologist*)).tw,kf
or/22–24
14 and 21 and 25
26 not (exp animals/not humans.sh)
limit 27 to yr=“1975-Current”
